# Baicalein and baicalin inhibit colon cancer using two distinct fashions of apoptosis and senescence

**DOI:** 10.18632/oncotarget.24015

**Published:** 2018-01-08

**Authors:** Jie Dou, Zhou Wang, Leon Ma, Bo Peng, Ke Mao, Chengqin Li, Mengqi Su, Changlin Zhou, Guangyong Peng

**Affiliations:** ^1^ State Key Laboratory of Natural Medicines, School of Life Science and Technology, China Pharmaceutical University, Nanjing 210009, P. R. China; ^2^ Division of Infectious Diseases, Allergy and Immunology and Department of Internal Medicine, Saint Louis University School of Medicine, Saint Louis, MO 63104, USA

**Keywords:** baicalein, baicalin, Scutellaria baicalensis georgi, colon cancer, apoptosis

## Abstract

Baicalein and baicalin are active components of the *Scutellaria baicalensis* Georgi and both have broad anti-tumor activity. However, how and whether baicalein and baicalin inhibit colon cancer is unclear. Here we demonstrate that baicalein and baicalin can significantly inhibit human colon cancer cell growth and proliferation. Furthermore, both can induce cell cycle arrest, and suppress cancer cell colony formation and migration. The suppressive effects are mechanistically due to the induction of colon cancer cell apoptosis and senescence mediated by baicalein and baicalin, respectively. Furthermore, we revealed that baicalin-induced senescence in tumor cells is due to its inhibition of telomerase reverse transcriptase expression in tumor cells, and that MAPK ERK and p38 signaling pathways are causatively involved in the regulation of colon cancer cell apoptosis and senescence mediated by baicalein and baicalin. In addition, our *in vivo* studies using human colon cancer cells in humanized mouse xenograft models, further demonstrated that baicalein and baicalin can induce tumor cell apoptosis and senescence, resulting in inhibition of tumorigenesis and growth of colon cancer *in vivo*. These data clearly suggest that baicalein and baicalin have potent anti-cancer effects against human colon cancer and could be potential novel and effective target drugs for cancer therapy.

## INTRODUCTION

Colorectal cancer (CRC) is one of the most common cancers in the USA and worldwide. Although significant progress has made in diagnosis and treatment, it is still the 3rd major cause of cancer death with 100, 000 new cases and 50, 000 cases estimated deaths a year [1–3]. Understanding the cancer pathogenesis and development of novel and effective drugs are still challenging issues in colon cancer. Given that failure in chemotherapy is due to the cytotoxicity and drug resistance of the frequently-used chemotherapeutic agents [4], more attention has been paid to the natural compounds that developed from botanical sources [5–8]. Actually, many promising drugs have been developed based on the natural products and applied in clinical trials for various disease treatments including cancers [9–11]. A better understanding of biological functions and mechanism of those natural compounds on cancer will provide novel targets for the clinical therapy against colon cancer and other cancers as well.

Flavonoids are natural polyphenols found in many fruits and vegetables, which are involved in various physiological and pathological processes and have been demonstrated to provide health benefits in humans. Baicalein (5, 6, 7-trihydroxyflavone), a phenolic flavonoid compound, is an active component of *Scutellaria baicalensis Georgi*, which has been widely used as a traditional Chinese herbal medicine for many disease treatments, including in inflammation, infectious diseases and anti-oxidation [12–16]. Baicalin, baicalein's conjugate, baicalein-7-glucuronide, also can be found in *Scutellaria baicalensis* [17]. Increasing evidence suggests that both baicalein and baicalin have strong anti-tumor effects in various cancers, including in breast cancer, prostate cancer, pancreatic cancer, esophageal squamous cell carcinoma and burkitt lymphoma [18–22]. Their anti-tumor mechanisms could involve induction of cancer cell apoptosis and activation of PI3K/AKT, mTOR and NF-KB signaling pathways [18–22]. However, limited information is known about how and whether baicalein and baicalin inhibit colon cancer. Furthermore, the molecular action mediated by baicalin against cancer is poorly understood. Extensive research on the inhibitory activities and mechanisms mediated by baicalin and baicalein, and comparisons of their difference on different types of cancers will be beneficial to evaluate their druggability.

Cellular senescence is a biological process by which normal diploid cells cease to divide and undergo growth arrest, but remain viable, metabolically active and possess unique transcriptional profiles and gene regulation signatures [23, 24]. There are two major categories of cellular senescence: (1) Replicative senescence (telomere-dependent senescence) [23, 24]; and (2) Premature senescence (extrinsic senescence) is induced by a variety of extrinsic forms of stress, such as oxidative stress, DNA damage, and activation of certain oncogenes, as well as some inflammatory cytokines and chemokines [25–28]. In addition to the most somatic cells undergoing aging or infected with age-related pathologies [29], cellular senescence is now thought to be a tumor suppressive mechanism that could be harnessed for cancer therapy [26, 30]. We have also recently discovered that both human tumor cells and regulatory T cells (Treg) can induce responder effector T cells into senescent T cells [31–34]. Improved understanding of molecular mechanisms for the generation of senescent cells and their molecular regulations will open new avenues to design novel vaccines and/or therapies for cancer.

In our current study, we explored the anti-tumor effects and related mechanisms mediated by baicalein and baicalin on human colon cancer. We observed that both baicalein and baicalin can significantly inhibit human colon cancer cell growth and proliferation, induce cell cycle arrest, and suppress cancer cell colony formation and migration. These suppressive effects are mechanistically due to the induction of colon cancer cell apoptosis and senescence. Importantly, we further demonstrated that baicalein and baicalin can induce tumor cell apoptosis and senescence, resulting in inhibition of tumorigenesis and growth of colon cancer *in vivo* in human colon cancer models. Our studies collectively suggest that baicalein and baicalin could be potential novel and effective target drugs for colon cancer therapy.

## RESULTS

### Baicalein and baicalin significantly inhibit human colon cancer cell growth and proliferation

**Figure 1:**
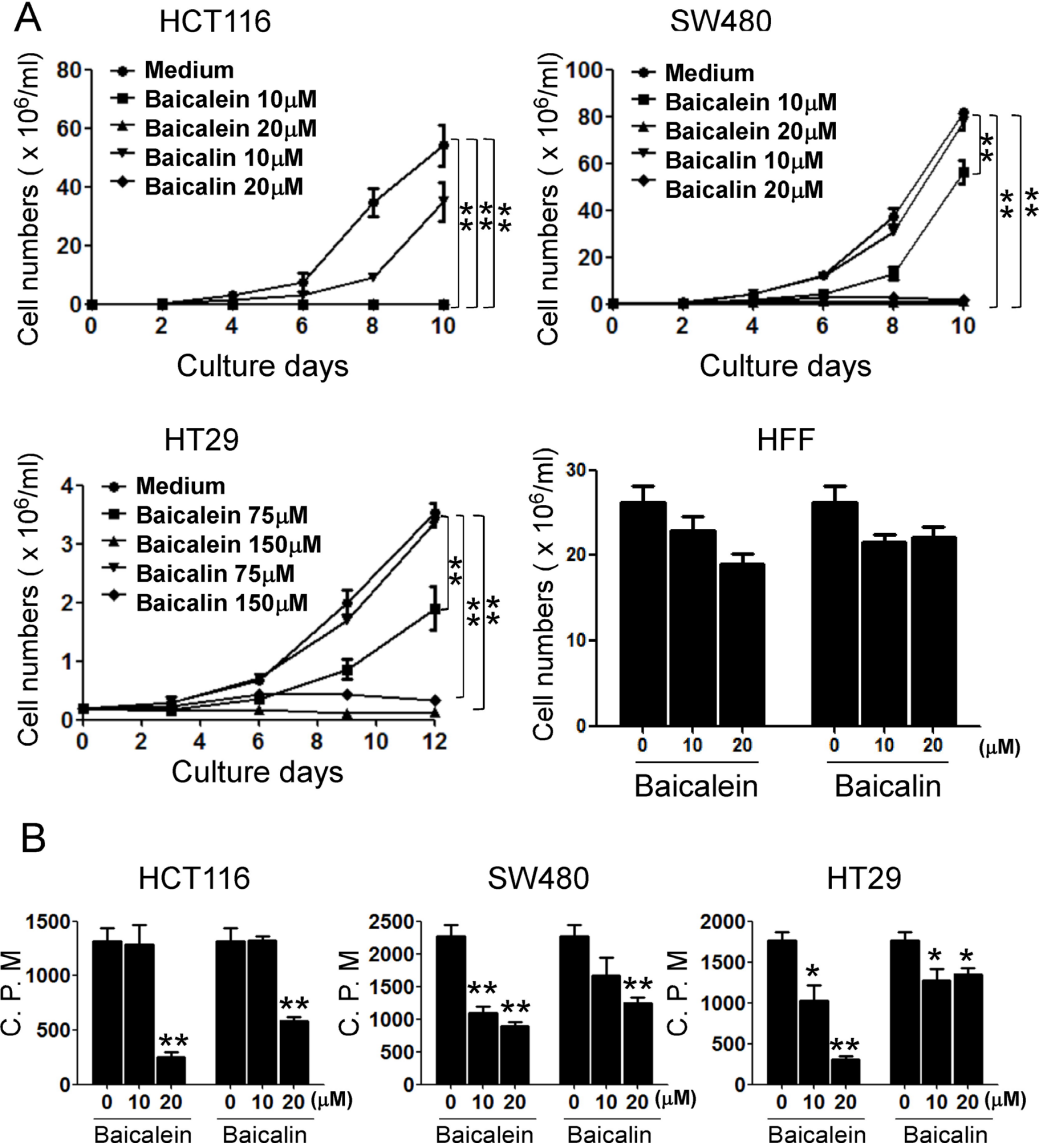
Baicalein and baicalin inhibit colon cancer cell growth and proliferation Three colon cancer cell lines (HCT116, SW480 and HT29) were cultured at a started number of 2 × 10^5^/well in 6-well plates, or 5 × 10^3^/well in 96-well plates, and treated with the indicated concentrations of baicalein or baicalin. The cell growth was evaluated at different time points using cell number counting (in **A**), and cell proliferation was determined using [^3^H]-thymidine assays (in **B**). Human foreskin fibroblast HFF cells treated with baicalein or baicalin for 72 h were included as a normal cell control (in A). Data shown in (A) and (B) are mean ± SD from three independent experiments with similar results. ^*^p < 0.05 and ^**^p < 0.01 compared with the medium control group.

Increasing evidence suggests that baicalein has strong capacity to inhibit tumor growth in various cancers [18–22]. Therefore, we reasoned that baicalein and baicalin may also directly influence colon cancer cell growth. To test this possibility, three human colon cancer cell lines were cultured in the presence of various concentrations of baicalein and baicalin, and tumor cell growth and proliferation were determined using cell growth curve and [^3^H]-thymidine incorporation assays. We observed that both baicalein and baicalin strongly inhibited tumor growth and proliferation of HCT116, HT29 and SW480 cells, which were in the dose-dependent inhibition manners (Figure 1A and 1B). However, both baicalein and baicalin didn't show an obvious inhibitory activity on human foreskin fibroblast (HFF) cell growth (Figure 1A), suggesting that baicalein and baicalin might specifically target tumor cells rather than normal cells. Notably, suppressive activity of baicalein on colon cancer growth is much stronger than that of baicalin. We found that low concentration of baicalin did not have inhibition on SW480 and HT 29 cell growth (Figure 1A). In addition, after 10 days of treatment, baicalein with both doses (20 and 50 μmol/l) nearly completely destroyed HCT116 cells, while most HCT116 cells remained their integrity in the presence of same concentrations of baicalin (Data not shown).

### Baicalein and baicalin block cell cycle progression of human colon cancer cells

**Figure 2:**
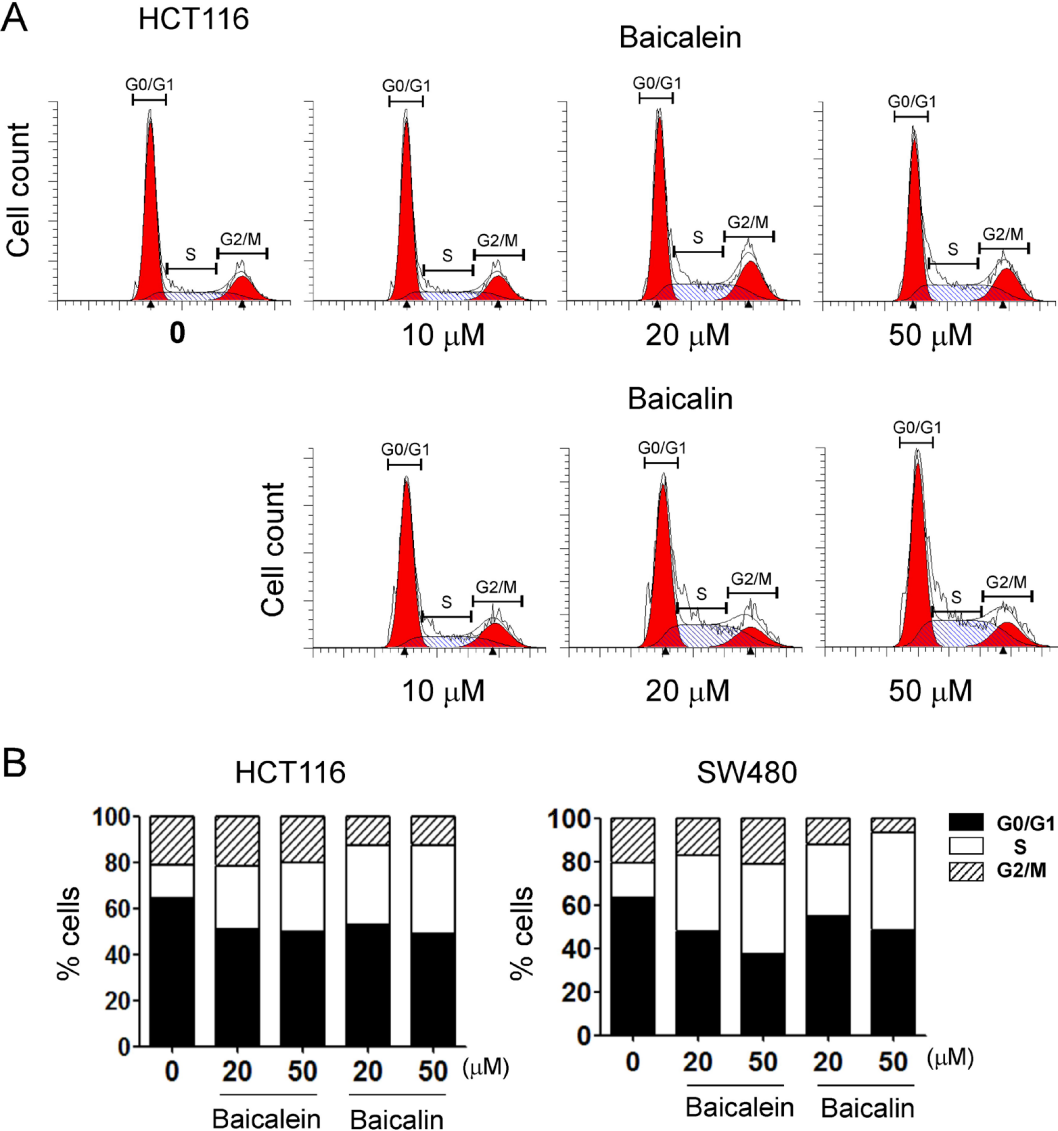
Baicalein and baicalin significantly promote colon cancer cell cycle arrest in S phase and decrease in G0/G1 phase (**A**) and (**B**) HCT116 and SW480 colon cancer cells were cultured for 72 h in the presence of the indicated concentrations of baicalein or baicalin. Cell cycle distribution in tumor cells was analyzed after incubation with 10 μg/ml propidium iodide and 100 μg/ml RNase A. Data are representative of three independent experiments with similar results.

Given significant inhibition of colon cancer cell growth and proliferation mediated by both baicalein and baicalin, we next determined whether baicalein and baicalin can alter human colon cancer cell DNA synthesis and cell cycle progression. We measured DNA content of colon cancer cells treated with different concentrations of baicalein and baicalin using flow cytometry analyses. As shown in Figure 2, treatment with both baicalein and baicalin significantly induced cancer cells to arrest in S phase and decrease in G0/G1 phase in all human colon cancer cell lines, especially in the high dose treatment groups. These results collectively indicated that baicalein and baicalin not only suppress colon cancer cell growth but also inhibit cell proliferation and cell cycle progression.

### Baicalein and baicalin inhibit colon cancer cell colony formation and migration

**Figure 3:**
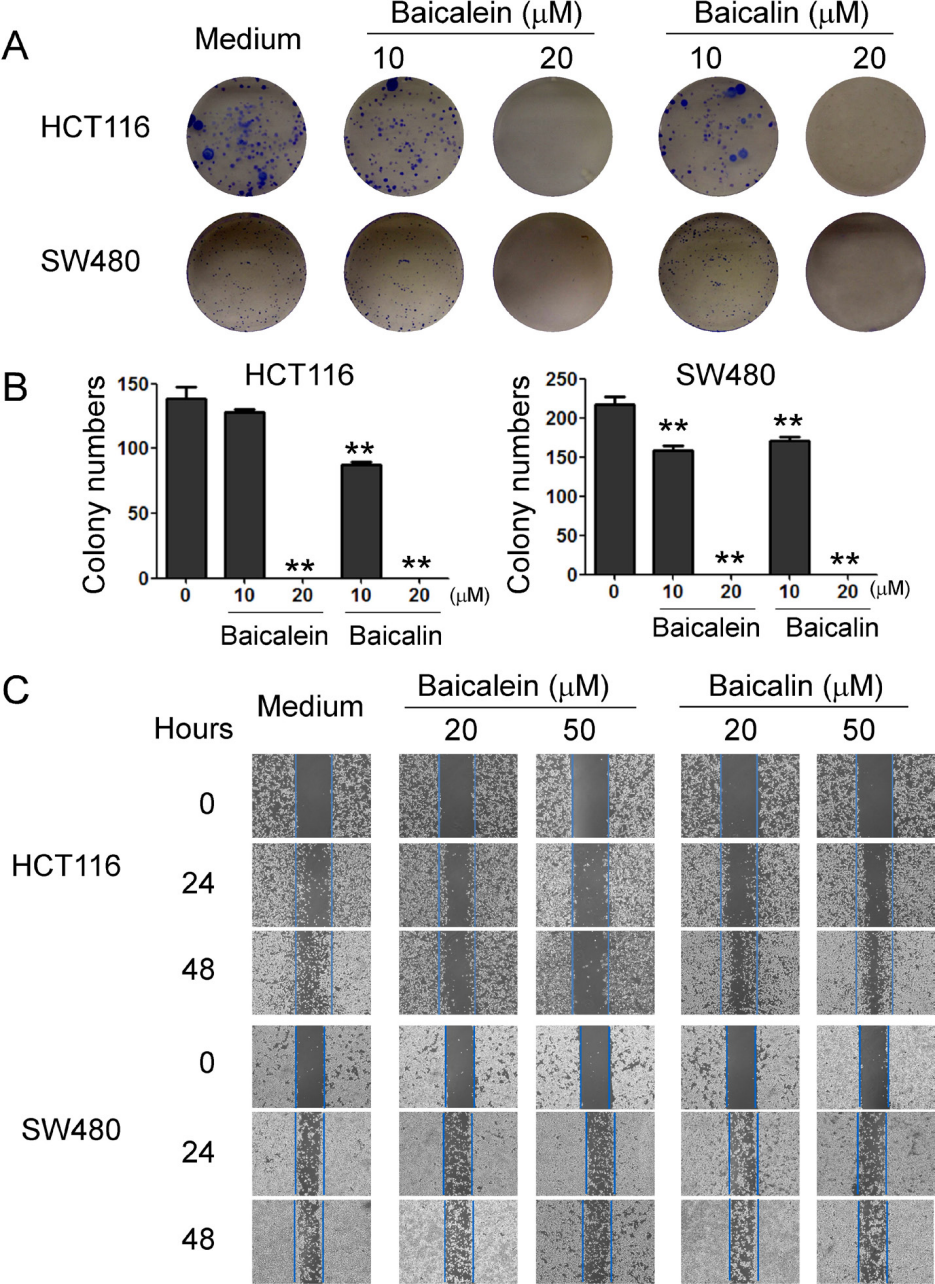
Baicalein and baicalin inhibit colon cancer cell colony formation and migration (**A**) and (**B**) Baicalein and baicalin treatments dramatically decreased the numbers and sizes of tumor colonies in HCT116 and SW480 cells after 2 weeks of culture. Two hundred to five hundred per well of colon cancer cells pre-treated with baicalein or baicalin, were seeded in 6-well plates for culture, and cell colonies counted after 10-14 days of culture. Results shown in the histogram (in B) are summaries of mean ± SD from three independent experiments. ^*^p < 0.05 and ^**^p < 0.01 compared with the medium control group. (**C**) Baicalein and baicalin treatments in HCT116 and SW480 colon cancer cells significantly inhibited the migration of tumor cells compared with the medium control group in the wound closure assays. Data shown are representative from three independent experiments with similar results.

In addition to determine cancer cell growth inhibition, we further investigated the functional role of baicalein and baicalin in regulating other key biological behaviors of colon cancer cells, including colony formation and migration capacities. As expected, we observed that the numbers and sizes of cell colonies were significantly decreased in HCT116 and SW480 cells after treatment with baicalein or baicalin (Figure 3A and 3B). Treatment with both baicalein and baicalin in the concentration of 20 μM almost completely inhibited the cell colony formation of HCT116 and SW480 cells. In addition, both baicalein and baicalin can markedly inhibit the colon cancer cell migration using the wound healing assay. However, the inhibitory effect of baicalin on colon cancer cell migration is not as obvious as that of baicalein (Figure 3C). Furthermore, HCT116 cells were more sensitive to both baicalein and baicalin than SW480 cells based on this migration assay. These results further suggested that baicalein and baicalin directly control cell growth and biological behaviors in colon cancer.

### Baicalein and baicalin induce colon cancer cell apoptosis and senescence

**Figure 4:**
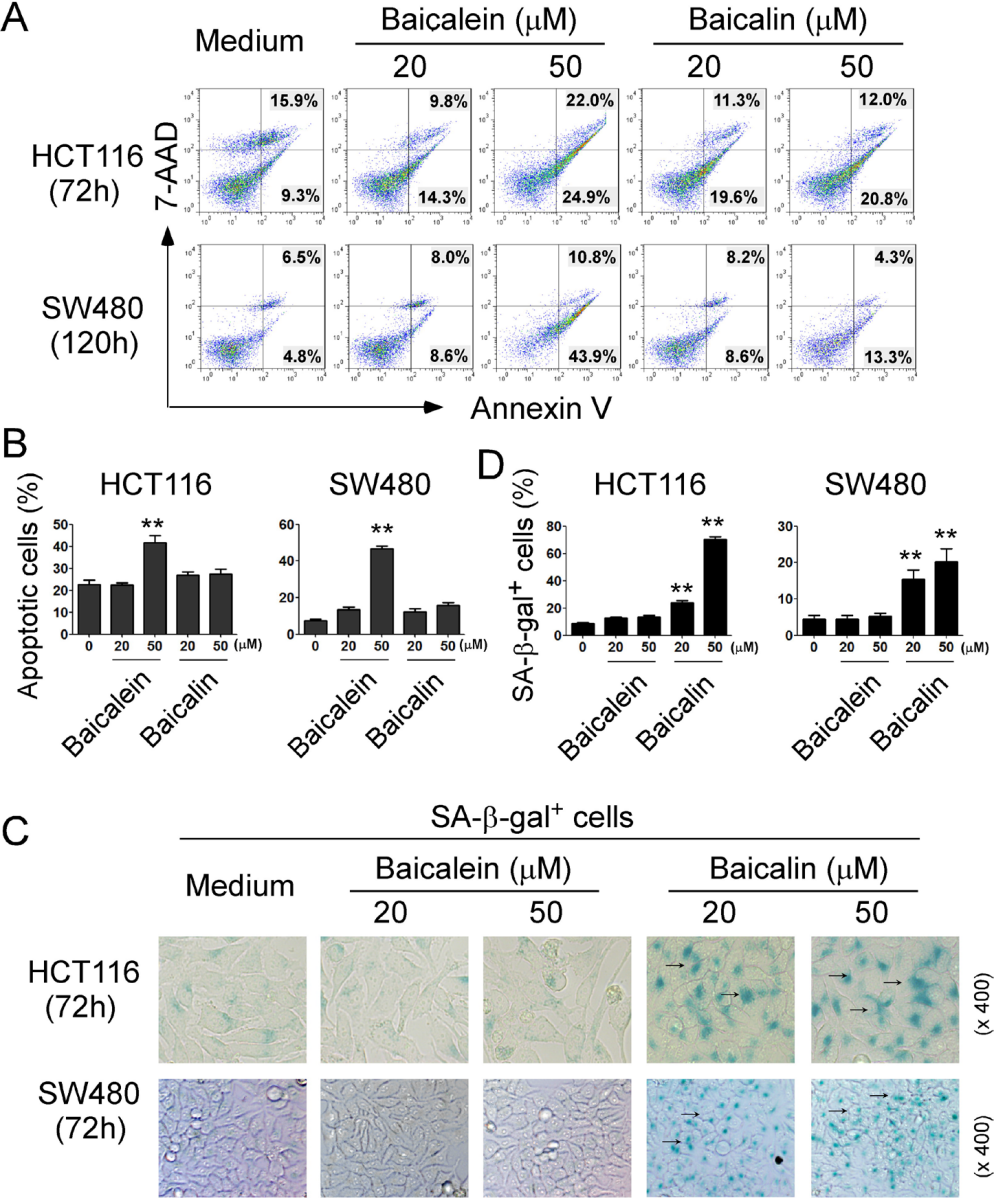
Baicalein- and baicalin-mediated inhibition of colon cancer cell growth and proliferation is due to induction of cell apoptosis and senescence, respectively (**A**) and (**B**) Significantly increased apoptotic cell populations were induced in HCT116 and SW480 cells after treatment with baicalein but not baicalin. (**C**) and (**D**) Treatment with baicalin but not with baicalein in HCT116 and SW480 cancer cells markedly induced SA-β-Gal positive cell populations in tumor cells. Tumor cells were cultured in the presence of indicated concentrations of baicalein and baicalin for 72 h or 120 h. Apoptosis in treated tumor cells was analyzed after staining with PE-labeled Annexin V and 7-AAD (in A). Senescent cells were analyzed using the SA-β-Gal activity assay and the SA-β-Gal positive cells were identified with dark blue granules as indicated by the arrows (in C). Data in (B) and (D) are mean ± SD from three independent experiments with similar results. ^*^p < 0.05 and ^**^p < 0.01 compared with the medium control group.

Suppression of tumor cell proliferation and growth mediated by baicalein and baicalin could be due to the induction of apoptosis or cytolysis in the tumor cells. Recent studies have shown that baicalein and baicalin can induce cancer cell apoptosis in pancreatic cancer, esophageal squamous cell carcinoma and burkitt lymphoma [20–22]. Therefore, we first measured apoptosis and cell death in colon cancer cells treated by baicalein or baicalin. We found that cultured with medium, both HCT116 and SW480 colon cancer cells contained some apoptotic cells (around 25% in HCT116 and 10% in SW480). Furthermore, consistent with the previous reports in other types of cancer cells, treatment with baicalein in 50 μM significantly induced tumor cell apoptosis in both HCT116 and SW480 cells (Figure 4A and 4B) [20–22]. Surprisingly, baicalin treatment did not markedly induce increased apoptosis or cell death in either colon cancer cell lines (Figure 4A and 4B), suggesting that suppression of human colon cancer cell growth and proliferation mediated by baicalein and baicalin may through the different molecular mechanisms.

Besides the induction of cell apoptosis, senescent human cells have permanent growth arrest, which could occur due to telomere shortening or a DNA damage response [23, 24]. We therefore reasoned that induction of senescence might be the mechanism involved in the suppressed cell growth and proliferation mediated by baicalin in colon cancer cells. In addition to cell cycle arrest and morphologic characteristics, SA-β-Gal is the first biomarker used to identify senescent human cells [31, 32]. We observed that culture with baicalin significantly increased the numbers of SA-β-Gal^+^ cells in HCT116 and SW480 colon cancer cells, indicating the induction of tumor cell senescence (Figure 4C and 4D). In contrast, treatment with baicalein in these tumor cells did not induce increased SA-β-Gal expression (Figure 4C and 4D). Our results collectively suggest that baicalein and baicalin treatment in colon cancer cells can induce both cell apoptosis and senescence respectively, resulting in the inhibition of cancer cell growth and functional behaviors.

### Baicalin but not baicalein inhibits telomerase reverse transcriptase, resulting in colon cancer cell senescence

**Figure 5:**
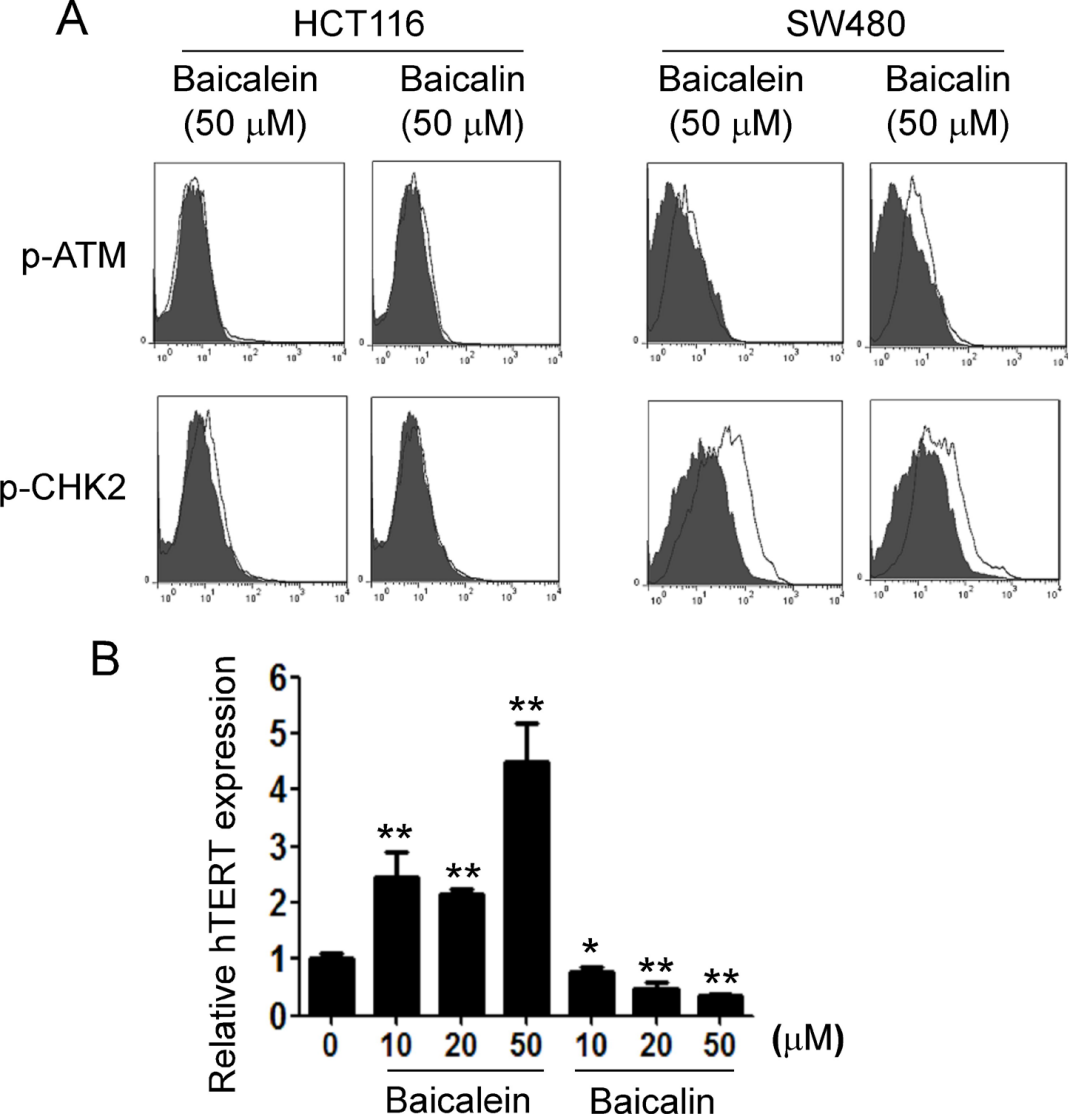
Baicalin, but not baicalein, significantly suppresses hTERT expression in colon cancer cells (**A**) Both baicalin and baicalein did not induce phosphorylated activation of ATM and its associated molecule CHK2 in colon cancer cells. HCT116 cells and SW480 cells were treated with baicalein or baicalin at indicated concentration for 72 h. The p-ATM and p-CHK2 expression in treated colon cancer cells were analyzed by the flow cytometry. Results shown are a representative of three experiments with similar results. (**B**) HCT116 cells were treated with baicalein or baicalin at indicated concentration for 24 h. mRNA in cells was purified for quantitative PCR analysis of hTERT expression. mRNA levels in each group were normalized to the relative quantity of GAPDH expression and then adjusted to hTERT levels in medium group (set as 1). Results shown in the histogram are mean ± SD from three independent experiments. ^*^p < 0.05 and ^**^p < 0.01 compared with the medium control group.

We next determined how baicalin induces senescence in tumor cells. The induction of DNA damage is the key molecular process in both apoptotic and senescent cells. The nuclear kinase ataxia-telangiectasia mutated protein (ATM) is the chief inducer of the DNA-damage response. We thus determined whether induction of ATM-associated DNA damage is the main trigger for baicalin-induced senescence in human colon cancer cells [38]. However, we found that both baicalein and baicalin did not induce significant activation and phosphorylation of ATM in HCT116 and SW480 cancer cells (Figure 5A). Furthermore, they also did not activate the other key DNA damage response protein, such as downstream target checkpoint kinase 2 (CHK2), in HCT116 and SW480 tumor cells during their induction of apoptosis and senescence [24]. These data provide the strong evidence that suppression of colon cancer growth and proliferation mediated by baicalein and baicalin is not due to the induction of DNA damage in cancer cells.

We then tested the other possibility that telomere shortening and/or dysfunction, a well-studied mechanism responsible for replicative senescence, may be involved in baicalin-induced senescence in colon cancer cells [23, 24]. We compared human telomerase reverse transcriptase (hTERT) expression in tumor cells treated with baicalein or baicalin for 24 hours. As expected, we observed that treatment with baicalin significantly down-regulated the expression level of hTERT in tumor cells. In contrast, treatment with baicalein dramatically promoted hTERT expression (Figure 5B). These results suggest that suppression of colon cancer growth and proliferation mediated by baicalein and baicalin is due to the different mechanisms in cancer cells, and that replicative senescence induction might be causatively related to baicalin-mediated senescence in colon cancer cells.

### MAPK ERK and p38 signaling pathways are involved in the regulation of colon cancer cell apoptosis and senescence induced by baicalein and baicalin

**Figure 6:**
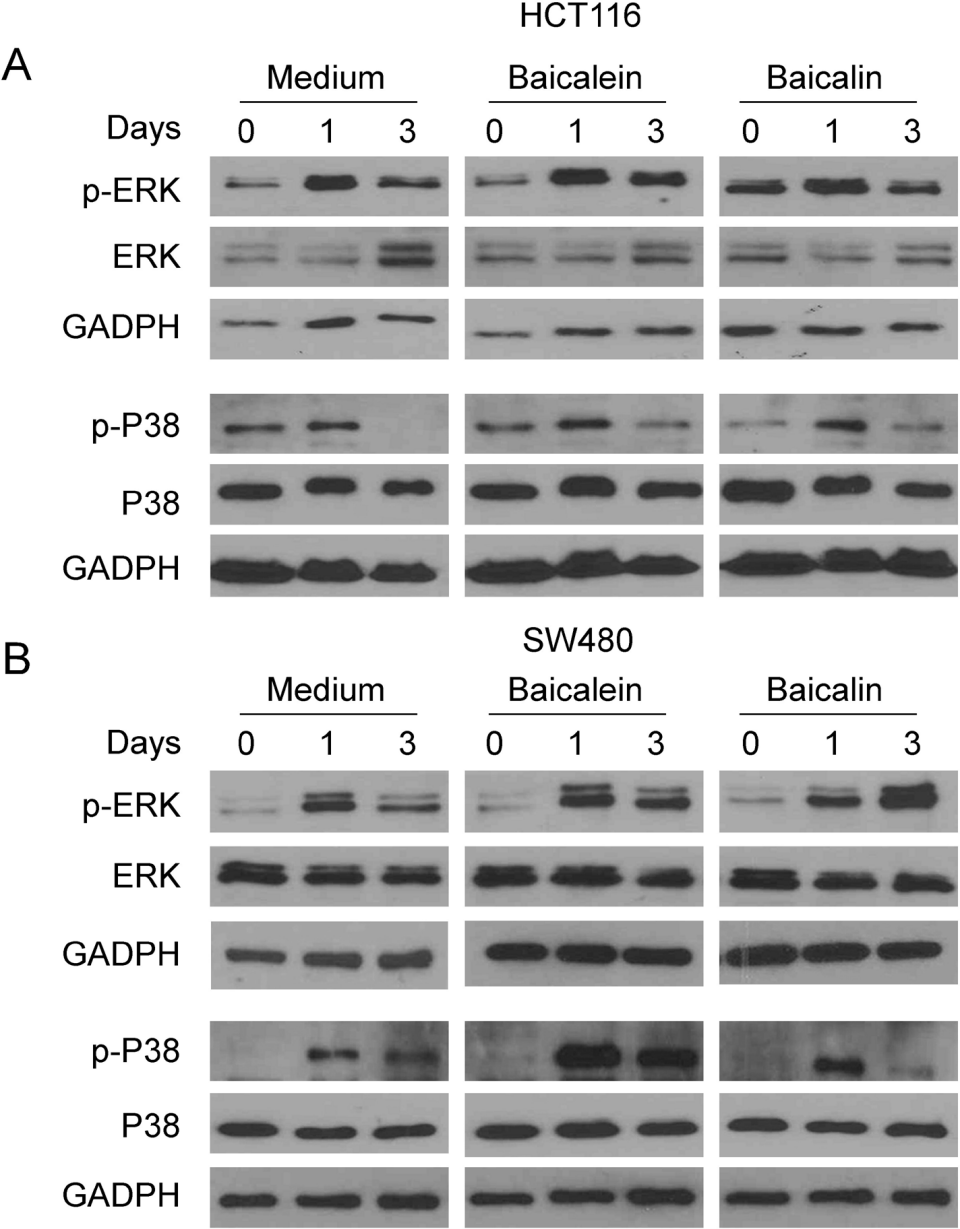
MAPK p38 and ERK1/2 signaling pathways involve colon cancer cell apoptosis and senescence induced by baicalein and baicalin (**A**) and (**B**) Both baicalein and baicalin treatment induced phosphorylation of ERK and p38 in HCT116 and SW480 tumor cells. Colon cancer cells were cultured in the presence of 50 μM *baicalein* and *baicalin* for the indicated times and cell lysates were prepared for western blot analyses.

The Mitogen-activated protein kinase (MAPK) pathway is related with many physiological effects, including apoptosis, cell proliferation, and senescence. MAPK signaling pathways play a major role in regulating cell cycle re-entry and oncogenic ras-induced senescence [39–42]. We have recently further shown that human Treg cell treatment selectively modulates ERK1/2 and p38 signaling pathways in targeted responder T cells that control the process of T cell senescence [31, 32]. We next explored whether induction of colon cancer cell cycle S arrest and both cell apoptosis and senescence mediated by baicalein and baicalin are involved in MAPK signaling modulation. We determined the activation of MAPKs, including ERK1/2, p38 and JNK in HCT116 and SW480 colon cancer cells treated with baicalein and baicalin using western blot analyses. We found that treatment with both baicalein and baicalin in HCT116 and SW480 colon cancer cells selectively activated ERK1/2 and p38, but not JNK, resulting in significantly enhanced phosphorylation of ERK1/2 and p38 in both colon cancer lines (Figure 6A and 6B, and data not shown). Our results indicated baicalein and baicalin can induce selective modulation of specific MAPK p38 and ERK1/2 signaling pathways in human colon cancer cells that might control the molecular processes of tumor cell cycle arrest, apoptosis and senescence.

### Baicalein and baicalin inhibit tumorigenesis and growth *in vivo*

**Figure 7:**
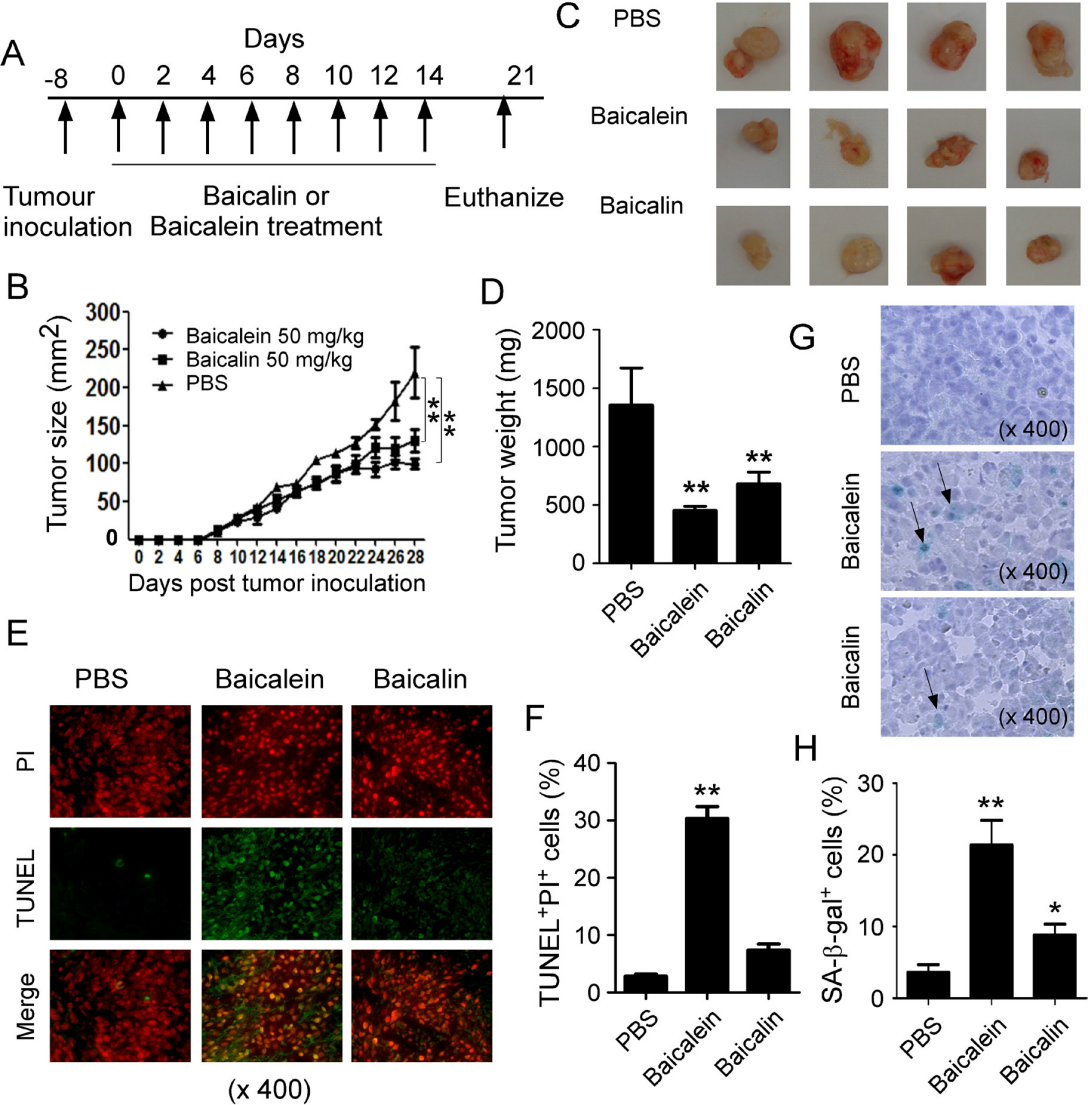
Baicalein and baicalin inhibit tumor growth and development *in vivo* in a colon cancer xenograft model (**A**) Experimental scheme for tumorigenesis studies with a colon cancer xenograft model. HCT116 cells (5 × 10^6^/mouse) were subcutaneously injected into NSG mice. After 8 days post tumor injection, the tumor-bearing mice were administrated with baicalein (50 mg/kg) or baicalin (50 mg/kg), and solvent control through intraperitoneal injection, respectively, at every other day for 2 weeks. (**B**) Both *baicalein* and *baicalin* dramatically inhibited HCT116 tumor growth in NSG immunodeficient mice. Tumor volumes were measured and presented as mean ± SD (n = 4 mice per group). P values were determined by the one-way analysis of variance (ANOVA). Similar results were obtained in three repeated experiments. (**C**) Representative image of the xenograft tumors obtained from the indicated groups at the endpoint of the experiments (day 29). (**D**) Treatments with both baicalein and baicalin had much lower tumor weight compared with that of solvent control group. Results shown are mean ± SD of the xenograft tumor weights from the indicated groups in the model at the endpoint of the experiments (day 29) (n= 4 mice per group). ^**^p < 0.01, compared with the solvent control group using unpaired *t*-test. (**E**) and (**F**) Increased apoptotic cells were observed in tumor tissues from the treatment group of baicalein but not baicalin in NSG mice. Cell apoptosis in the paraffin-embedded tissues was analyzed by the TUNEL and PI staining at the endpoint of experiment. Panel in (E) is photomicrographs of the TUNEL and PI staining in sections of tumor tissues from different treatment groups. Panel in (F) is the mean ± SD of percentages of apoptotic cells (TUNEL^+^PI^+^ cells) in the total PI^+^ cells in the tumor tissues from 4 mice of each group. ^**^p < 0.01, compared with the control treatment mice using unpaired *t*-test. (**G**) and (**H**) Large amounts of senescent tumor cells were observed in tumor tissues from both treatments of baicalein and baicalin in NSG mice. SA-β-Gal expression was determined in the tumor frozen tissues from different groups at the endpoint of experiment. Panel in (G) is photomicrographs of SA-β-Gal expression in tumor tissues from different groups. Panel in (H) is the mean ± SD of percentages of SA-β-Gal^+^ cells per high microscope field (× 400) in the tumor tissues from 4 mice of each group. ^**^p < 0.01, compared with the control treatment mice using unpaired *t*-test.

Our *in vitro* studies clearly identified the mechanism and molecular signaling of baicalein and baicalin in suppressing human colon cancer tumor cell growth and proliferation. We next performed complementary *in vivo* studies, using human colon cancer cells in humanized NOD-scid IL2Rγ^null^ (NSG) mouse xenograft models, and explored whether baicalein and baicalin can inhibit tumorigenesis and growth of colon cancer *in vivo* [35–37, 43]. HCT116 cells were subcutaneously injected into NSG mice. After 8 days post tumor injection (tumor size reached around 5 × 5 mm), baicalein and baicalin (50 mg/kg) were administered through intraperitoneal injection into the tumor-bearing mice, respectively, at every other day for 2 weeks. Tumor growth was evaluated. At the end of experiments (Day 29), tumors were isolated from different groups of the sacrificed mice and weighted (Figure 7A). HCT116 tumor cells administered with PBS control grew progressively in NSG mice. However, treatment with both baicalein and baicalin significantly inhibited tumor growth (Figure 7B). Furthermore, tumor sizes collected from the baicalein or baicalin treated HCT116 groups were much smaller than those in the PBS treatment group (Figure 7C). In addition, the average tumor weight obtained from either the baicalein treatment group or baicalin treatment group also showed much lower than that of PBS control group (Figure 7D). In addition to the tumor growth, we verified the induction of tumor cell apoptosis and senescence mediated by baicalein and baicalin treatments in tumor tissues. Consistent with the *in vitro* results, we found that baicalein but not baicalin treatment dramatically induced tumor cell apoptosis in HCT116 tumor tissues (Figure 7E and 7F). Furthermore, baicalin treatment significantly induced tumor cell senescence in the tumor tissues evidencing by the increased SA-β-Gal^+^ cell populations (Figure 7G and 7H). Unexpectedly, we also observed that baicalein administration exhibited much stronger activity in senescence induction in HCT116 tumor tissues, which is different from our *in vitro* observations showing that baicalein did not induce HCT116 tumor cell senesce in co-culture system (Figure 4). Collectively, these results clearly suggest that both baicalein and baicalin directly inhibit tumor growth and tumorigenesis in human colon cancer, and that induction of tumor cell apoptosis and/or senescence is critical suppressive fashions.

## DISCUSSION

*Scutellaria baicalensis Georgi* is one of the most important traditional Chinese medicines, which is widely used for the disease treatments in China, Korea and Japan. Improved understanding of its active components and anti-tumor mechanisms against different types of cancers will provide novel strategies for cancer treatment. In the current study, we explored anti-tumor activities and molecular mechanisms on human colon cancer cells mediated by baicalin and baicalein, two key components of *Scutellaria baicalensis* Georgi. Our studies clearly demonstrated that both baicalein and baicalin can significantly inhibit human colon cancer cell growth and biological functions *in vitro*, as well as prevent tumorigenesis *in vivo* in a humanized mouse xenograft tumor model. We further identified that their suppressive effects are mechanistically due to the induction of colon cancer cell apoptosis or/and senescence, causatively involving down-regulated hTERT expression and MAPK ERK and p38 signaling activation. Our studies indicate that baicalein and baicalin could be potential novel and effective therapeutic drugs for human colon cancer.

Increasing evidence suggests that baicalein and baicalin have strong anti-tumor effects in various cancers [18–22], but little is known about their suppressive effect on colon cancer. In our effect to explore this important issue, we discovered that both baicalein and baicalin have strong anti-tumor activity against human colon cancer. Importantly, our current studies further identified the molecular mechanisms responsible for the tumor suppression mediated by baicalein and baicalin. Consist to the previous studies in other types of cancers of pancreatic cancer and esophageal squamous cell carcinoma, we raveled that baicalein can induce colon cancer cell apoptosis [20, 22]. However, we did not observe any obviously increased apoptosis in colon cancer cells mediated by baicalin, but it strongly induced tumor cell senescence. Cellular senescence was initially described in human fibroblasts with limited passage capacity in cell culture [44]. Senescent cells remain viable, metabolically active and possess unique transcriptional profiles and gene regulation signatures [23, 24], which is found in most somatic cells undergoing aging or infected with age-related pathologies [29]. Our recent studies suggest that induction of effector T cells senescence is a novel suppressive mechanism mediated by tumor cells and tumor-associated regulatory T cells within the tumor suppressive microenvironment [31–34]. In addition, cellular senescence is now thought to be a tumor suppressive mechanism that could be harnessed for cancer therapy [26, 30]. Our current studies further indicate that induction of colon cancer senescence could be a novel anti-tumor mechanism mediated by baicalin. In support of this notion, our *in vivo* studies in a humanized NSG mouse xenograft tumor model showed that both baicalein and baicalin can strongly induce tumor cell senescence, resulting in inhibition of tumorigenesis and growth of colon cancer *in vivo.* Notably, we unexpectedly observed that baicalein could also induce tumor cell senescence *in vivo* different from our *in vitro* results. This pheromone strongly suggested their mutual conversion between baicalein and baicalin *in vivo* [45]. Baicalein can be hydrolyzed into baicalin by the UDP-glucuronosyltransferase (UGT), and baicalin can also be reduced to baicalein by glucuronidase (GUS). Furthermore, baicalin is the main metabolite of baicalein through various routes of medication [45]. Collectively, these results dissect potential novel mechanisms utilized by baicalein and baicalin for anti-tumor effect, and should open new avenues to develop novel alternative therapeutic strategies for cancer treatments.

Our current studies also address the key question of what molecular processes are involved in the induction of apoptosis and senescence induced by baicalein and baicalin. We clearly showed that inhibition of hTERT might be the cause for baicalin-mediated senescence in tumor cells, further suggesting induction of replicative senescence in tumor cells. In addition, we did not observe significant changes of DNA damage response in colon cancers induced by both baicalein and baicalin. These studies suggest that effects on colon cancer cells mediated by baicalein and baicalin are through different molecular mechanisms. Further studies will be focused on the dissection of potential mechanism responsible for baicalein-induced apoptosis in colon cancer cells. The focuses include other extrinsic forms of stress on colon cancer cells mediated by baicalein, such as oxidative stress, and activation of certain oncogenes, as well as some inflammatory cytokines and chemokines, which could be the causes of cell apoptosis and/or senescence [25–28]. In addition, we have shown that induction of apoptosis and senescence mediated by baicalein and baicalin involves specific regulatory signaling pathways. MAPK signaling pathways have been shown to play a major role in regulating cell cycle re-entry and oncogenic ras-induced senescence [39–42, 46]. Our recent studies have also demonstrated that human Treg cells selectively modulate the specific MAPK p38 and ERK1/2 signaling pathways in responder T cells, which controls responder T cell senescence [31–34]. Our current studies indicate that MAPK ERK and p38 signaling pathways are involved in baicalein and baicalin induced apoptosis and senescence. Studies from other groups have shown that many other signaling pathways are also involved in the regulation of cancer cell apoptosis mediated by baicalein and baicalin, including PI3K/AKT, mTOR and NF-kb signaling pathways [18–22]. In addition, the current study did not consider the KRAS/NRAS/BRAF mutation statuses in colon cancer cells which are important for clinical outcomes and survival in cancer patients [47, 48]. Therefore, continued efforts will include the identification of the molecularly regulatory mechanisms and causative links among these signaling pathways involved in anti-tumor effects mediated by baicalein and baicalin.

Taken together, our studies identify that baicalein and baicalin can significantly inhibit human colon cancer *in vitro* and *in vivo*. We further revealed that their anti-tumor effects were mechanistically due to the induction of colon cancer cell apoptosis or/and senescence. These data clearly suggest that both baicalein and baicalin have potent anti-cancer effects against human colon cancer and are potential novel and effective target drugs for cancer therapy.

## MATERIALS AND METHODS

### Chemical compounds

Baicalein (Purity 98.5%) and baicalin (Purity 91.5%) were purchased from the Kanghua Company (Nanjing, Jiangsu, China) and were dissolved in dimethyl sulfoxide (DMSO, Sigma, St. Louis, USA). A 50 mM stock solution were prepared and stored in –20°C for the experiments.

### Human cell lines

Human colon cancer cell lines (HCT116, HT29 and SW480) and human foreskin fibroblast (HFF) were purchased from the American Type Culture Collection (ATCC, Manassas, VA, USA). These cells were maintained in DMEM medium containing 10% fetal calf serum (FCS).

### Cell growth and proliferation assay

Colon cancer cell lines and HFF cells were cultured at a started number of 2 × 10^5^/well in 6-well plates in the presence of different concentrations of baicalein and baicalin in triplicate wells. Cell growth was evaluated at different time points with counting cell numbers. In addition, cell proliferation was determined using [^3^H]-thymidine incorporation assays as we previously described [31, 33, 35, 36]. In brief, different numbers of tumor cells (5 × 10^3^, 1 × 10^4^ or 2 × 10^4^) were cultured in 96-well plates in cell assay medium containing 2% FCS in the presence of different concentrations of baicalein and baicalin. After 56 hours of culture, [^3^H]-thymidine was added at a final concentration of 1 μCi/well, followed by an additional 16 hours of culture. The incorporation of [^3^H]-thymidine was measured with a liquid scintillation counter (PerkinElmer, Waltham, MA, USA).

### Cell cycle and apoptosis assays

Colon cancer cells were cultured for 72 or 120 hours in the presence of different concentrations of baicalein and baicalin, and apoptosis was analyzed after staining with PE-labeled Annexin V and 7-AAD (BD Biosciences, San Diego, CA, USA), as we previously described [31, 37]. For cell cycle analysis, treated tumor cells were fixed with 70% ethanol overnight, washed with PBS and incubated with propidium iodide (10 μg/ml) and RNase A (100 μg/ml). Untransfected cells served as a control. Stained cells were analyzed on a FACSCalibur (BD Bioscience) and the data were analyzed with FlowJo software (Tree Star, Ashland, OR, USA). For determination of cell apoptosis in tumor tissues, the terminal deoxynucleotidyl transferase (TdT)-mediated dUTP digoxigenin nick-end labelling (TUNEL) method (Vazyme, China) was utilized. Briefly, paraffin-embedded sections were deparaffinized with xylene and dehydrated, and then added proteinase K for 20 minutes. Slides were further stained with the FITC-labelled TdT and followed with the propidium Iodide (PI) (1 μg/ml) (cell nucleus labeling). The stained cells were examined and counted with a microscope (400 ×).

### Flow cytometry analysis

The expression of DNA damage response markers on tumor cells were determined by FACS analysis after staining with anti-human specific antibodies, including anti-phosphorylated CHK2, H2AX and ATM, and then secondary anti-rabbit antibody conjugated with either PE or FITC. These antibodies were purchased from Cell Signaling Technology or BD Biosciences. All stained cells were analyzed on a FACSCalibur flow cytometer (BD Bioscience) and data analyzed with FlowJo software (Tree Star).

### Senescence associated β-Galactosidase (SA-β-Gal) staining

Senescence associated β-Galactosidase (SA-β-Gal) activity in tumor cells was detected as we previously described [31–33]. Briefly, colon cancer cells were cultured for 3 days in the presence of different concentrations of baicalein or baicalin. Cells were fixed in 3% formaldehyde, and followed to incubate overnight at 37°C with freshly prepared SA-β-Gal staining solution (1 mg/ml X-gal, 5 mM K_3_Fe[CN]_6_, 5 mM K_4_Fe[CN]_6_, 2 mM MgCl_2_ in PBS at pH 6.0). The stained cells were washed with H_2_O and examined with a microscope.

### Western-blotting analysis

Colon cancer cells were cultured in the presence of the indicated concentrations of baicalein or baicalin for 0, 24 h, and 72 h. Whole cell lysates were prepared for western blotting. The antibodies used in western blotting are as follows: anti-ERK, anti-phospho-ERK, anti-p38, anti-phospho-p38, anti-JNK, anti-phospho-JNK, and anti-GAPDH rabbit polyclonal antibodies (Cell Signaling Technology, Danvers, MA, USA).

### Real-time PCR

Total RNA of tumor cells was extracted at 24 h after treatment with baicalein or baicalin using an RNeasy Mini kit (Qiagen) and then used for reverse transcription according to the manufacturer's instruction. The sequences of the primers for human telomerase reverse transcriptase (hTERT) are as follows: 5′ ATGCGACAGTTCGTGGCTCA 3′ (forward) and 5′ ATCCCCTGGCACTGGACGTA 3′ (reverse). The quantification of real-time PCR products was performed using QuantiFast Probe RT-PCR Kit (Qiagen). The housekeeping gene GAPDH was used as internal control for RNA integrity and normalization.

### Colony formation assay

Two hundred to five hundred per well of colon cancer cells pre-treated with baicalein or baicalin, were seeded in 6-well plates for culture. Cell colonies were fixed with 4% formaldehyde, stained with 0.5% Crystal Violet for 15 min at room temperature, and then counted after 10–14 days of culture [37].

### Wound healing assay

Colon cancer cells were plated in 6-well plates and grown to confluence. A wound area was generated by scraping cells with a 200 μl pipette tip across the entire diameter of the dish and extensively rinsed with the medium to remove all cellular debris. The scratches were photographed after additional 24 and 48 hours of culture in the presence of different concentrations of baicalein or baicalin. The closure was estimated as the wounded area relative to the initial area [37].

### *In vivo* tumorigenesis studies

NOD-scid IL2Rγ^null^ (NSG, 6–8 weeks) immunodeficient mice were purchased from The Jackson Laboratory and maintained in the institutional animal facility. All animal studies have been approved by the Institutional Animal Care Committee. For tumorigenesis studies, HCT116 cells (5 × 10^6^/mouse) were subcutaneously injected into NSG mice. After 8 days post tumor injection (tumor size reached around 5 × 5 mm), the tumor-bearing mice were randomly divided into 3 groups (*n* = 4) and administrated with baicalein (50 mg/kg), baicalin (50 mg/kg), and solvent control through intraperitoneal injection, respectively, at every other day for 2 weeks. Tumor size was measured with calipers every 2–3 days. Tumor volume was calculated on the basis of two-dimensional measurements. At the end of experiments (Day 29), the mice were sacrificed and tumors were isolated and weighted. Furthermore, tumor tissues were embedded into OCT and prepared for cryostat sections (4∼8 μm), and SA-β-Gal expression was assayed, as described above. In addition, paraffin-embedded tissues were prepared for cell apoptosis analyses with a TUNEL assay.

### Statistical analysis

Statistical analysis was performed with GraphPad Prism5 software. Data are expressed as mean ± standard deviation (SD). For multiple group comparison *in vivo* studies, the one-way analysis of variance (ANOVA) was used, followed by the Dunnett's test for comparing experimental groups against a single control. For single comparison between two groups, paired Student's *t* test was used. Nonparametric *t*-test was chosen if the sample size was too small and did not fit a Gaussian distribution.
